# Isolated rib fractures in geriatric patients

**DOI:** 10.4103/1817-1737.36552

**Published:** 2007

**Authors:** Elsayed M. Elmistekawy, Abd Almohsen M. Hammad

**Affiliations:** *Cardio-Thoracic Surgery, Faculty of Medicine, Tanta University, Tanta, Egypt*

**Keywords:** Blunt chest trauma, elderly, rib fractures

## Abstract

**INTRODUCTION:**

The goal of this study was to investigate the short-term outcomes in patients older than 60 years with isolated rib fractures and admitted to emergency hospital.

**MATERIALS AND METHODS:**

This study included patients who were 60 years old or more and sustained blunt chest injury and had isolated rib fractures. The following data were obtained from the medical records: age, gender, number of fracture ribs, side of fracture ribs, mechanism and nature of injury, preexisting medical conditions, complications, admission to intensive care unit (ICU), need for mechanical ventilation, length of ICU and hospital stay and mortality.

**RESULTS:**

For the study, 39 patients who were 60 years old or more and admitted to the hospital because of isolated rib fractures were enrolled. There were 28 males (71.7%) and 11 females (28.3%) with mean age of (66.84 ± 4.7) years. No correlation was found between comorbidities and hospital outcomes except in those who were diabetic (*P*-value = 0.005) and those with chronic lung disease (*P*-value = 0.006). Pulmonary complications were the most frequent complications encountered in those patients. Pulmonary complications were: lung contusion in 8 patients (20.5%) and pulmonary infection in 6 patients (15.8%).

**CONCLUSION:**

Elderly patients sustaining blunt chest trauma had significant morbidity and potential for mortality.

Elderly persons constitute an increasing proportion of patients admitted to trauma centers.[1] A recent review demonstrating that 10% of patients admitted to a level II trauma center had evidence of rib fractures; furthermore, the overall mortality for this group was 12%, with a 33% incidence of significant pulmonary complications.[2–3]

Rib fractures occur more commonly with increasing age, which usually associated with decreased bone density.[4] It was documented that morbidity and mortality are more with elderly patients compared to younger trauma patients.[3,5,6] The goal of this study was to investigate the short-term outcomes in patients older than 60 years with isolated rib fractures and admitted to trauma center and to examine morbidity and mortality in those patients and the relationship between comorbidities and number of rib fractures.

## Materials and Methods

### Patient population

This case series study was conducted in Cardiothoracic Surgery Department, Tanta Emergency Hospital. It included patients 60 or more years old that sustained blunt chest injury and had isolated rib fracture over 3 years' period. Patients with diagnosis other than isolated rib fractures or who had associated nonthoracic injuries were excluded from the study. Rib fractures were diagnosed clinically and confirmed radiologically.

### Study design

The following data were obtained retrospectively from the medical records: age, gender, number of fracture ribs, side of fracture ribs, mechanism and nature of injury, preexisting medical conditions, complications, admission to intensive care unit, need for mechanical ventilation, length of ICU and hospital stay, mortality.

### Statistical methods

Data were presented as mean ± standard deviation or number and percentage. Discrete data was analyzed using chi-square test. Multivariate linear regression models were done to examine the association between the demographic and clinical parameters with morbidity and mortality. ‘*P*’ value was considered significant when it was less than 0.05. Statistical analysis was done using SPSS program (SPSS 10 + Menu Tab, USA).

## Results

During the study period, 39 patients who were 60 years old or more admitted to the hospital because of isolated rib fractures were enrolled. There were 28 males (71.7%) and 11 females (28.3%). The mean age was (66.84 ± 4.7) years (average 60-79 years). Eighteen patients (46.1%) had left-sided fracture ribs, 17 patients (43.6%) had right-sided fracture ribs and 4 patients (10.3%) had bilateral fracture ribs. The fracture ribs was due to fall from a height in 23 patients, motor vehicle accident in 8 patients, animal trauma in 5 patients and due to other causes in 3 patients (Sport-related accidents, assault and impact of foreign objects). The average number of fractured ribs was 1.84 ± 0.7 ribs (range 1-8 ribs). Thirteen patients had single-rib fracture, 19 patients had multiple-rib fractures (2-4) while 7 patients had more than 4 ribs fractured.

The number of fractured ribs was associated with morbidity (*P*-value = 0.012) but not the mortality. There was no association between the side of rib fracture or mechanism of trauma and morbidity and mortality.

Concomitant comorbidities were found in 66.7% of patients [Table 1]. No association was found between comorbidities and hospital outcomes except in those who were diabetic (*P*-value = 0.005) and those with chronic lung disease (*P*-value = 0.006).

**Table 1:** Association of preexisting comorbidities and hospital outcomes

Comorbidity	No. (%) of patients	Morbidity		Mortality	
			
		No. (percentage)	*P* value	No. (percentage)	*P* value
Diabetes mellitus	9 (23.09)	7 (77.7)	0.005	1 (11.1)	NS
Hypertension	14 (35.89)	6 (42.56)	NS	1 (7.14)	NS
Chronic lung disease	6 (15.38)	4 (66.6)	0.001	3 (50)	0.006
Ischemic heart disease	6 (15.38)	2 (33.3)	NS	1 (16.6)	NS
Hepatic impairment	6 (15.38)	2 (33.3)	NS	2 (33.3)	NS
Neurological problems	4 (10.25%)	1 (25)	NS	1 (25%)	NS

NS = Non significant

Admission to ICU was needed in 10 patients (25.7%) for 1.64 ± 3.2 days (range 0-13 days); 3 of them needed mechanical ventilation. Length of hospital stay was 7.6 ± 4.2 days (range 3-21 days).

Pulmonary complications were the most frequent complications encountered in those patients [Table 2]. Pulmonary complications were lung contusion in 8 patients (20.5%) and pulmonary infection in 6 patients (15.8%). Other pulmonary complications were pneumothorax in 4 patients (10.2%), hemothorax in 3 patients (7.7%) and hemopneumothorax in 2 patients (5.1%). Significant association was found between pulmonary complications; and mortality (*P* = 0.015), which occurred in 4 patients; 3 of them died as a consequence of pulmonary infection and the fourth mortality was due to respiratory failure in a hepatic patient who required mechanical ventilation.

**Table 2:** Association of complications and mortality

	Mortality	
	
Complication	No. (percentage)	*P* value
Lung contusion	8 (20.51)	NS
Pulmonary infection	7 (17.49)	0.015
Need for mechanical ventilation	3 (7.69)	0.00
Hemothorax	4 (10.25)	NS
Pneumothorax	3 (7.69)	NS
Hemopneumothorax	2 (5.12)	NS

NS = Non significant

## Discussion

Elderly people who sustain blunt chest trauma have mortality and morbidity rates that are twice the corresponding rates prevalent among the younger population.[5]. Admission characteristics of our study population revealed that falls and vehicle crashes were the largest contributors to trauma admission in this group. Declining vision and hearing, preexisting medical condition, medications and alteration of judgment all probably contributed to some extent to their injuries.[7]

Increased morbidity and mortality in elderly patients with traumatic rib fractures are attributed to several factors such as anatomical difference between young and elderly people (osteoporosis, decreased muscle mass and thinned vertebral bodies), as well as decreased physiological reserve (low cardiopulmonary status and lower immunity),[8,9]. and more likelihood of presence of associated comorbidities. In a study by Morris *et al.,*[10] preexisting diseases including cirrhosis, coagulopathy, ischemic heart disease, chronic obstructive pulmonary disease and diabetes all significantly increased the morbidity and mortality in elderly trauma patients. Increased number of rib fractures was found to correlate with the morbidity in our patients. This is in agreement with Bulger *et al.,*[5] who found marked increase in the incidence of pneumonia (31%) and mortality (19%) in elderly patients with 3-4 rib fractures; those with more than 6 rib fractures had 33% mortality and 51% occurrence of pneumonia; and for each additional rib fracture in the elderly, mortality increased by 19% and was associated with 27% increase in the risk of pneumonia. They also found that the number of ventilation days, length of ICU stay and length of hospital stay also increased in those patients. Increase in the number of rib fractures correlated directly with increasing pulmonary morbidity and mortality. Patients sustaining fractures of 6 or more ribs were at significant risk of death from causes unrelated to the rib fractures.[11]

In agreement with our results, Stawicki *et al.,*[12] found that pulmonary complications were the most common complications in trauma patients with rib fractures. Acute respiratory failure, pleural effusion and pneumonia were more common in elderly patients compared to young patients. Increased age was found to be an independent risk factor for pneumonia in trauma patients.[13].

In the current study, chronic lung disease was found correlated with mortality; this explains the causes of mortality in this study, viz, respiratory failure and pulmonary infection. Preexisting lung problems lead to more frequent pulmonary complications and infection. A recent study demonstrated that elderly patients with multiple-fracture ribs and preexisting cardiopulmonary disease were at high risk of complications, ICU readmission and longer hospitalization.[14]. Pulmonary infection and need of mechanical ventilation were correlated with mortality in the current study. Mechanical ventilation reflects the severity of the injury and predisposes to atalectesis and pneumonia. Pneumonia was found to be an independent predictor of death in old patients with fractured ribs in Bergeron *et al.* study,[15] which reported 17.9% incidence of pneumonia in the studied patients. Pneumonia occurred more frequently in nonsurvivors (36.7%) compared to survivors (14.3%). Among the preadmission comorbiditities, only diabetes was found to be associated with increased morbidity (*P*=0.0005). This is in agreement with Barnea *et al.,*[16] who found that diabetes correlates significantly with morbidity in elderly patients with isolated rib fractures. Diabetic patients are more vulnerable to infections, have a slower healing process and lower immunological status compared to nondiabetic patients.

## Conclusion

Elderly patients who sustain blunt chest trauma have significant morbidity and potential for mortality. Aggressive management of these patients in the form of hospitalization, strict blood sugar control, adequate pain control and aggressive chest physiotherapy is required.
